# Supplementary material for: BMI1-Mediated Pemetrexed Resistance in Non-Small Cell Lung Cancer Cells Is Associated with Increased SP1 Activation and Cancer Stemness
Source: Cancers (Basel). 2020 Jul 27;12(8):2069. doi: 10.3390/cancers12082069 (PMC7463866; doi:10.3390/cancers12082069)
Supplement: Supplementary file 1 [file cancers-12-02069-s001.zip › Supplementary Materials_Cancers_202006.pdf]

Supplementary Materials

Figures

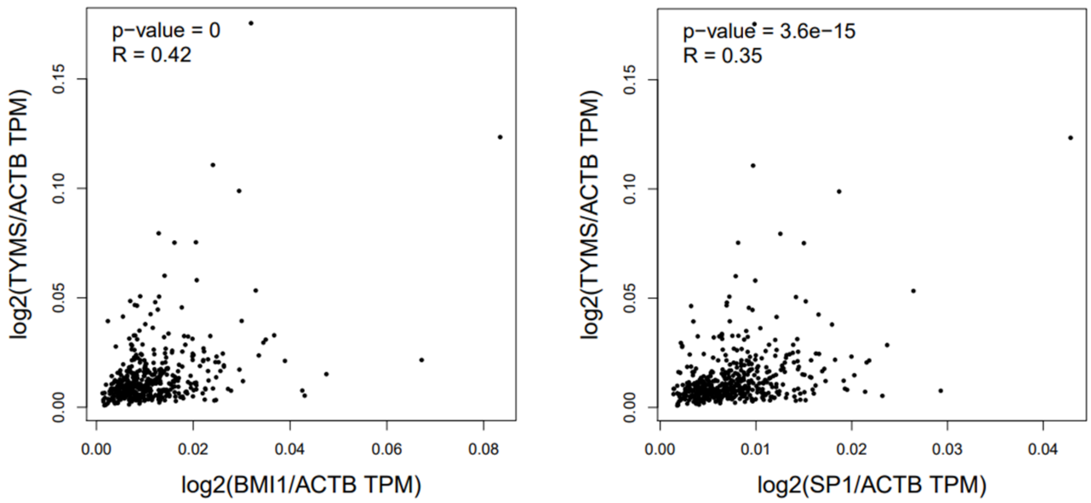

Figure S1. The positive correlation between BMI1/TYMS or SP1/TYMS was found in TCGA database. The mRNA expression levels of BMI1/SP1/TYMS among lung adenocarcinoma patients were obtained from TCGA database and analyzed by GEPIA website.

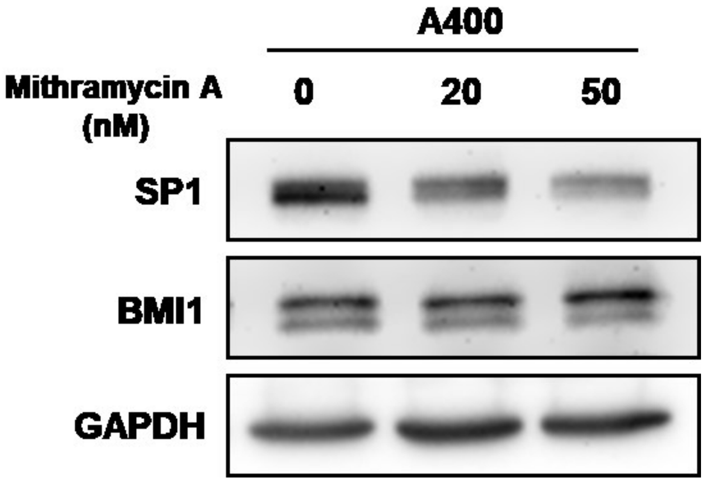

Figure S2. Inhibition of SP1 activity did not influence BMI1 expression in pemetrexed resistant NSCLC cells. Pemetrexed resistant A400 cells were treated with the indicated concentration of mithramycin A for 48 hours and the expression of SP1 or BMI1 was determined by western blot.

Tables

Table S1. Antibodies used in this study.

Target	Company	Catalog No.
BMI1	Novus Biologicals, Centennial, CO, USA	NBP1-33748
β-actin	Sigma-Aldrich, St. Louis, MO, USA	A5441
CD44		
E-cadherin	Santa Cruz Biotechnology, Inc., Dallas, TX, USA	sc-21791
Flag(M2)	Sigma-Aldrich, St. Louis, MO, USA	F1804
GAPDH	GeneTex International Corporation, HsinChu city, Taiwan.	GTX100118
Ki67	Abcam, Cambridge, MA, USA	ab16667
N-cadherin	Santa Cruz Biotechnology, Inc., Dallas, TX, USA	sc-59987
Notch1	Abcam, Cambridge, MA, USA	sb27526
Snail1	Cell Signaling Technology, Danvers, MA, USA	38795
thymidylate synthase	GeneTex International Corporation, HsinChu city, Taiwan.	GTX103235
α-tubulin	Proteintech Group, Inc., Rosemont, IL, USA	66031-1-Ig
Anti-Mouse IgG (HRP-linked)	PerkinElmer, Waltham MA, USA	NEF822001EA
Anti-Rabbit IgG (HRP-linked)	PerkinElmer, Waltham MA, USA	NEF812001EA
