# Supplementary material for: BMI1-Mediated Pemetrexed Resistance in Non-Small Cell Lung Cancer Cells Is Associated with Increased SP1 Activation and Cancer Stemness
Source: Cancers (Basel). 2020 Jul 27;12(8):2069. doi: 10.3390/cancers12082069 (PMC7463866; doi:10.3390/cancers12082069)
Supplement: Supplementary file 1 [file cancers-12-02069-s001.zip › Western blot whole images_R1_20200711.pdf]

Figure 1B

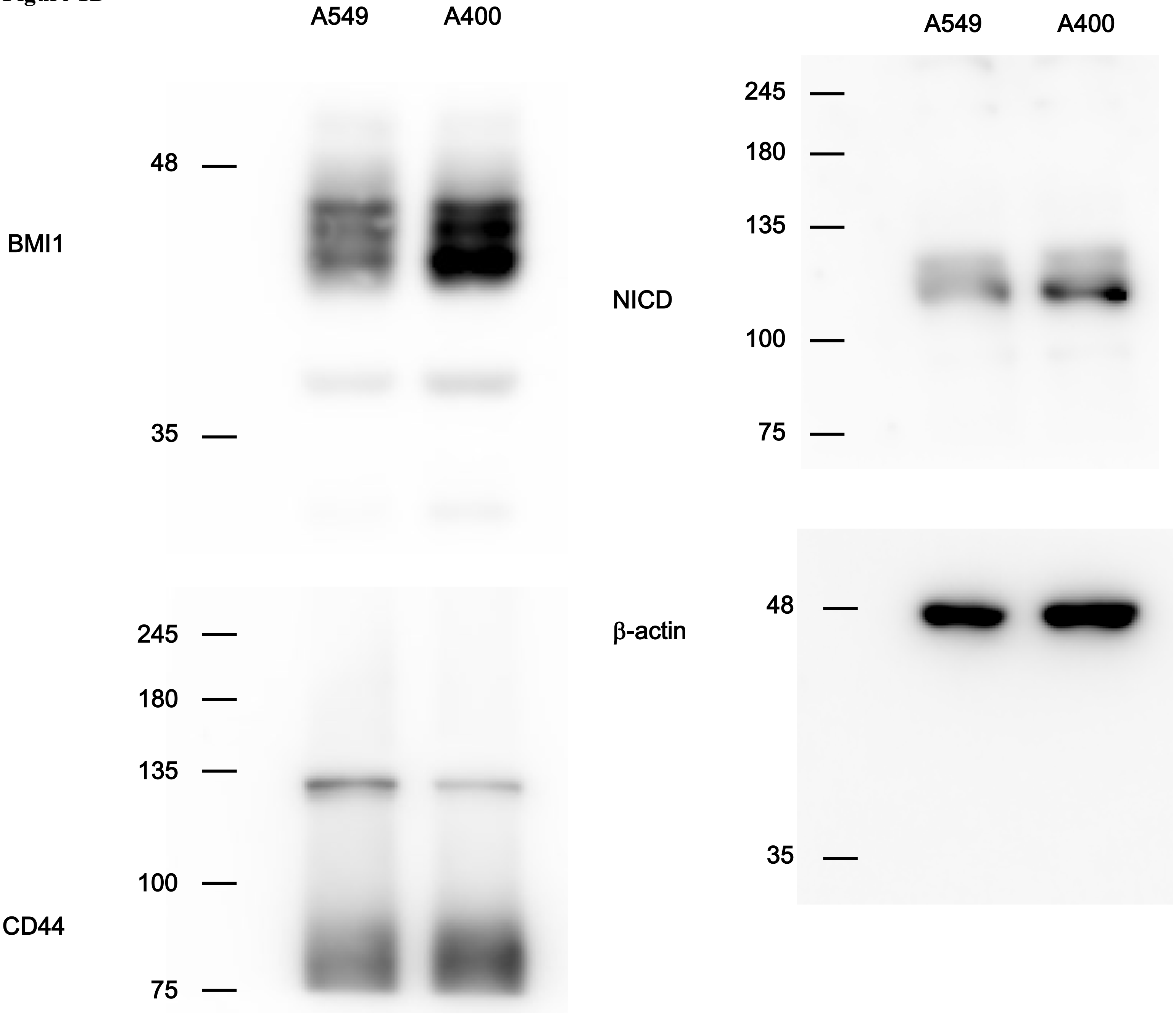
A549
A400
48
35
BMI1
245
180
135
100
75
CD44
A549
A400
245
180
135
100
75
NICD
48
35
β-actin

Figure 1C

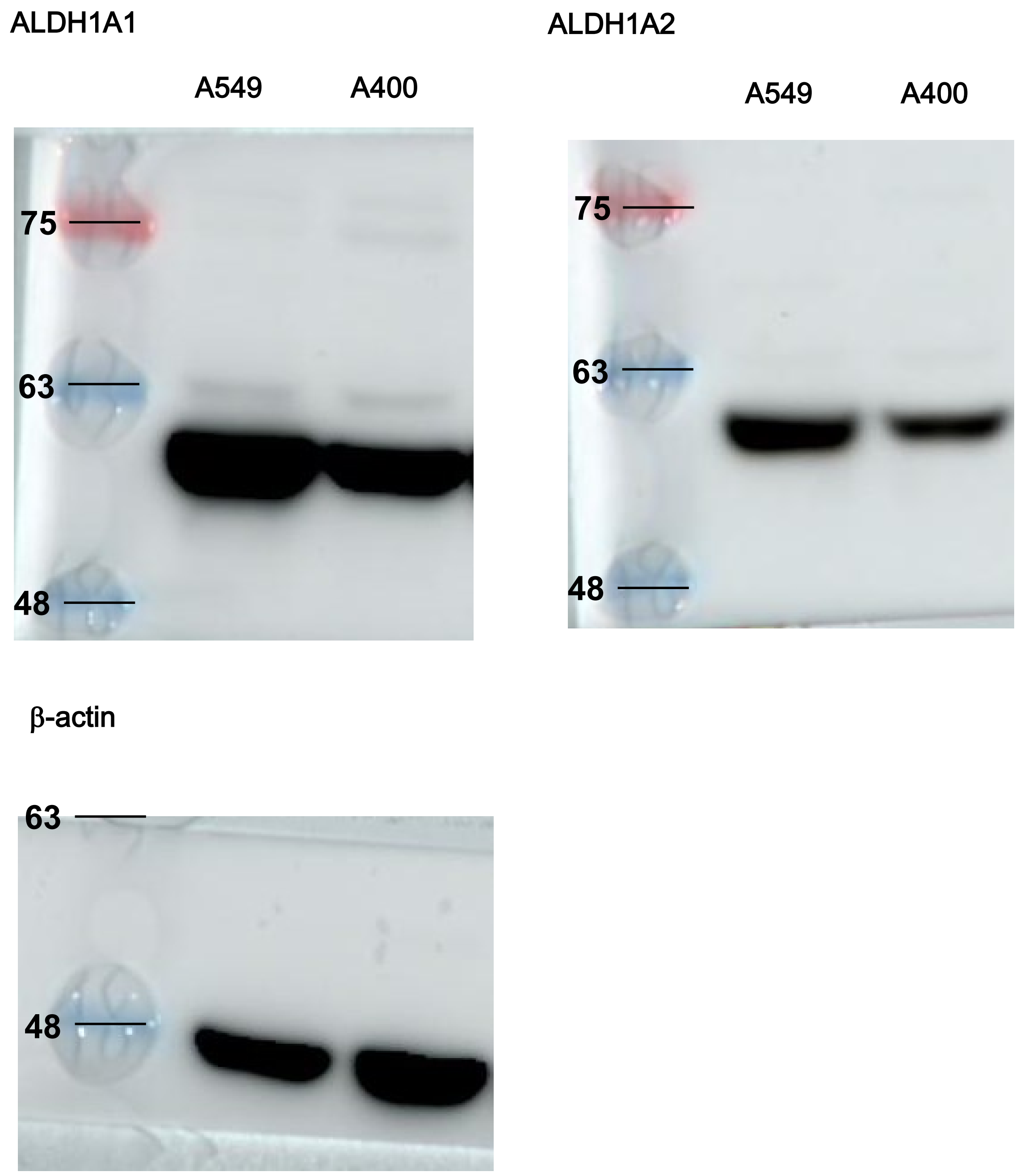
ALDH1A1
A549
A400
75
63
48
ALDH1A2
A549
A400
75
63
48
β-actin
63
48

Figure 1D

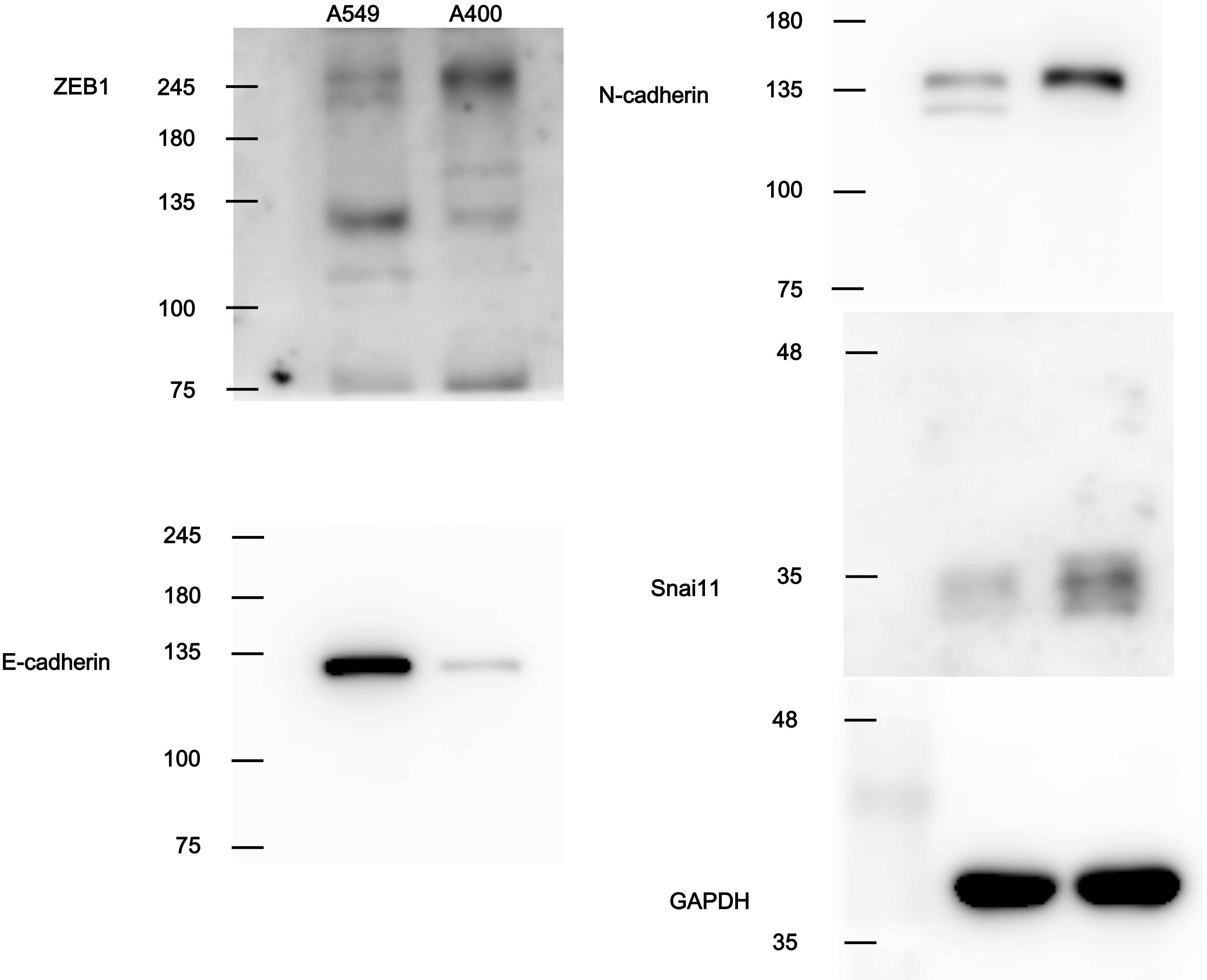
A549
A400
ZEB1
245
180
135
100
75
N-cadherin
180
135
100
75
E-cadherin
245
180
135
100
75
Snai11
48
35
GAPDH
48
35

Figure 2B

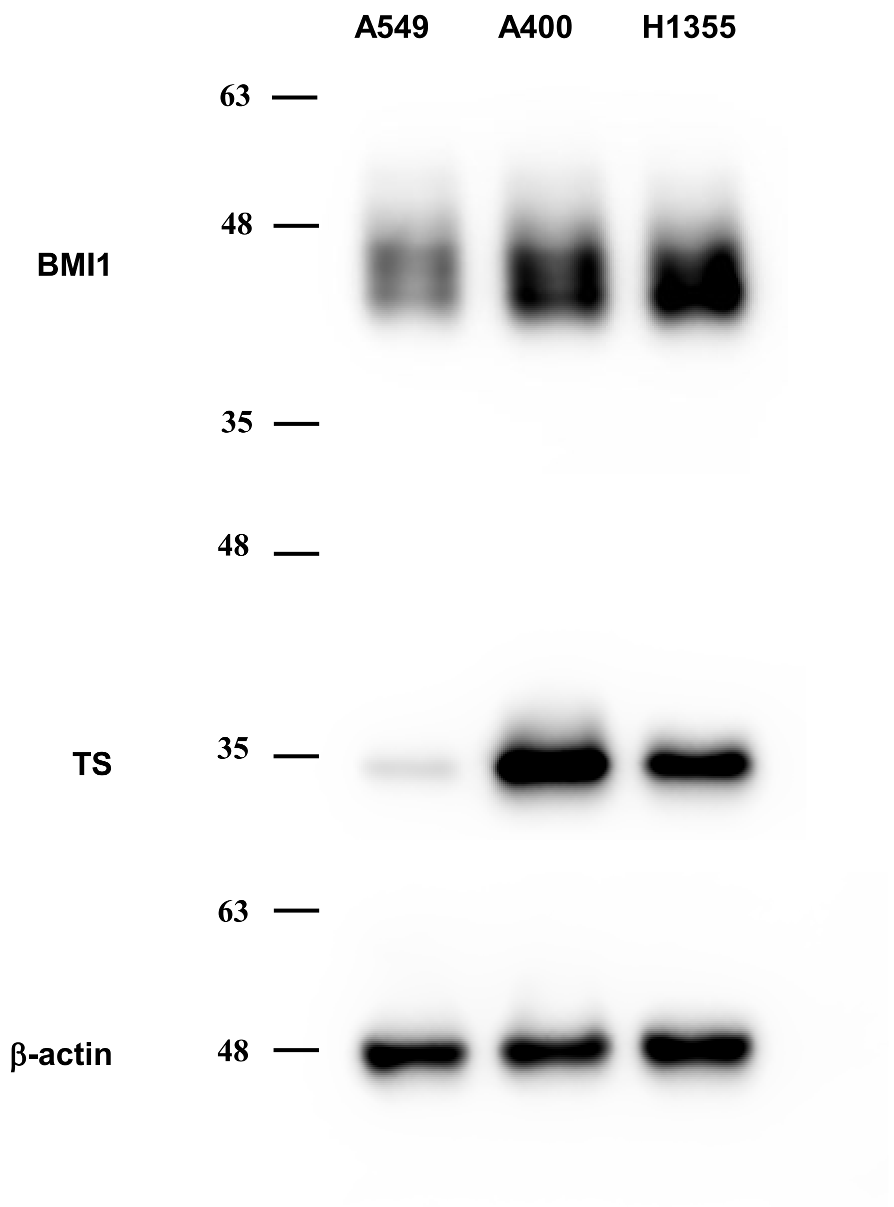

Figure 3A.

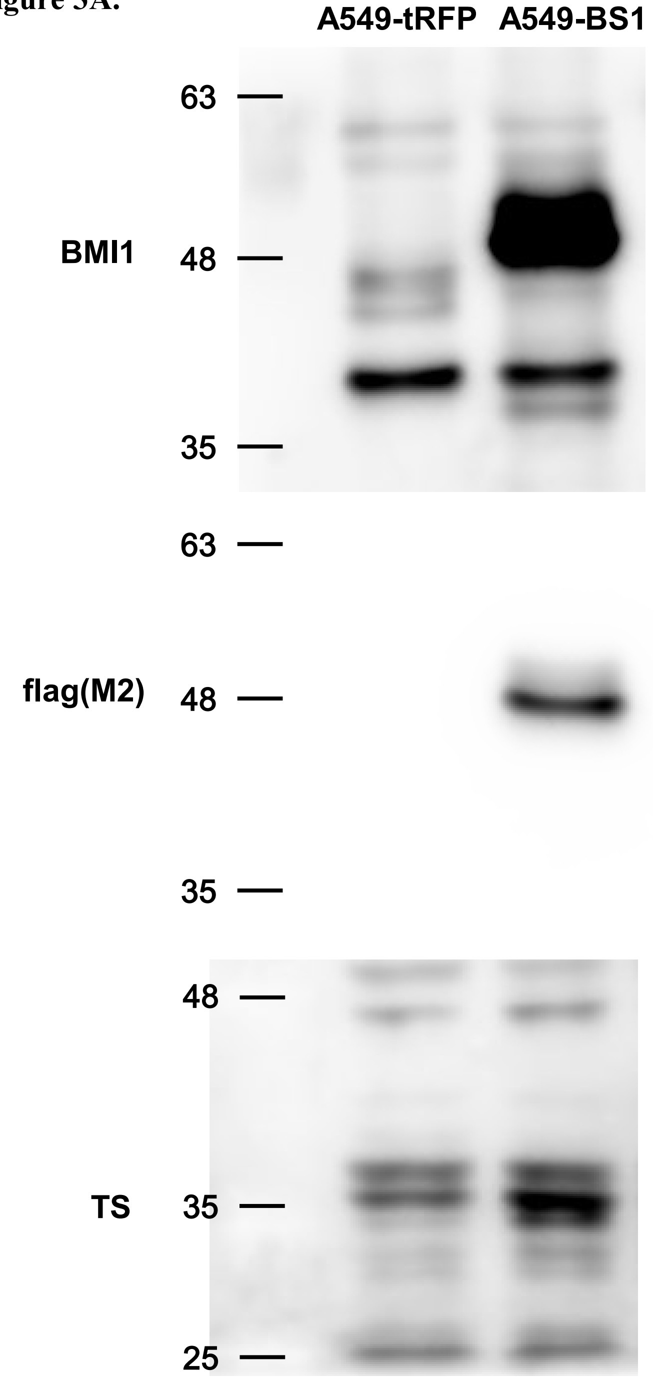

Figure 3C.

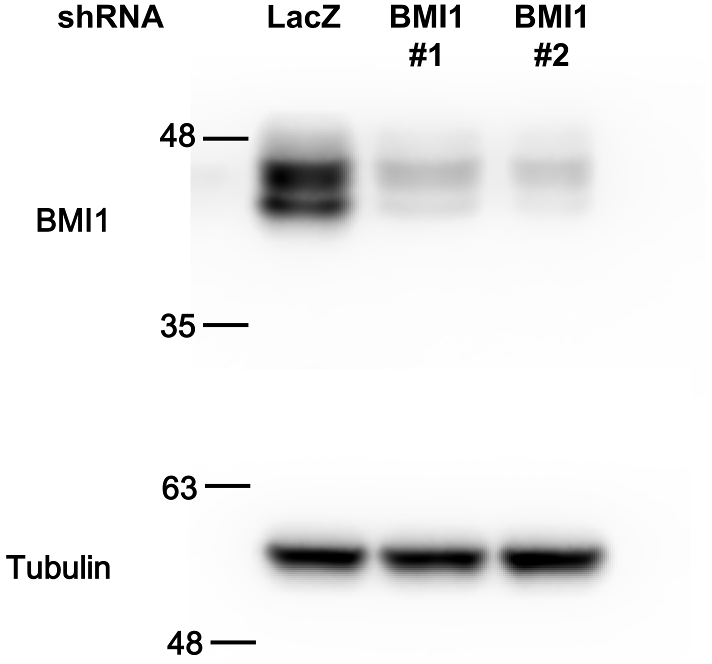

Figure 4A.

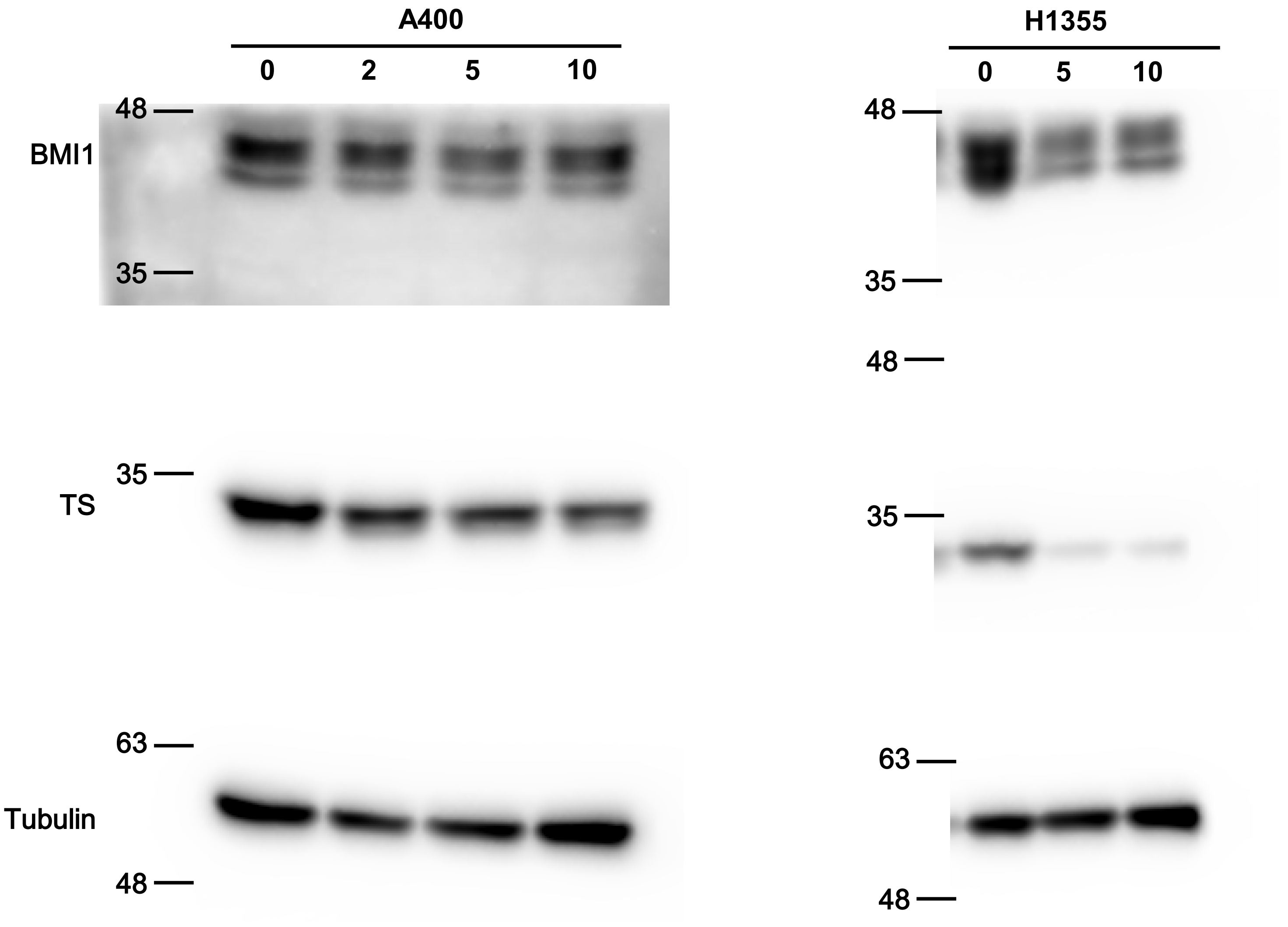

A400
0
2
5
10
H1355
0
5
10
48
BMI1
35
48
35
35
TS
35
63
Tubulin
48
63
48

Figure 4C.

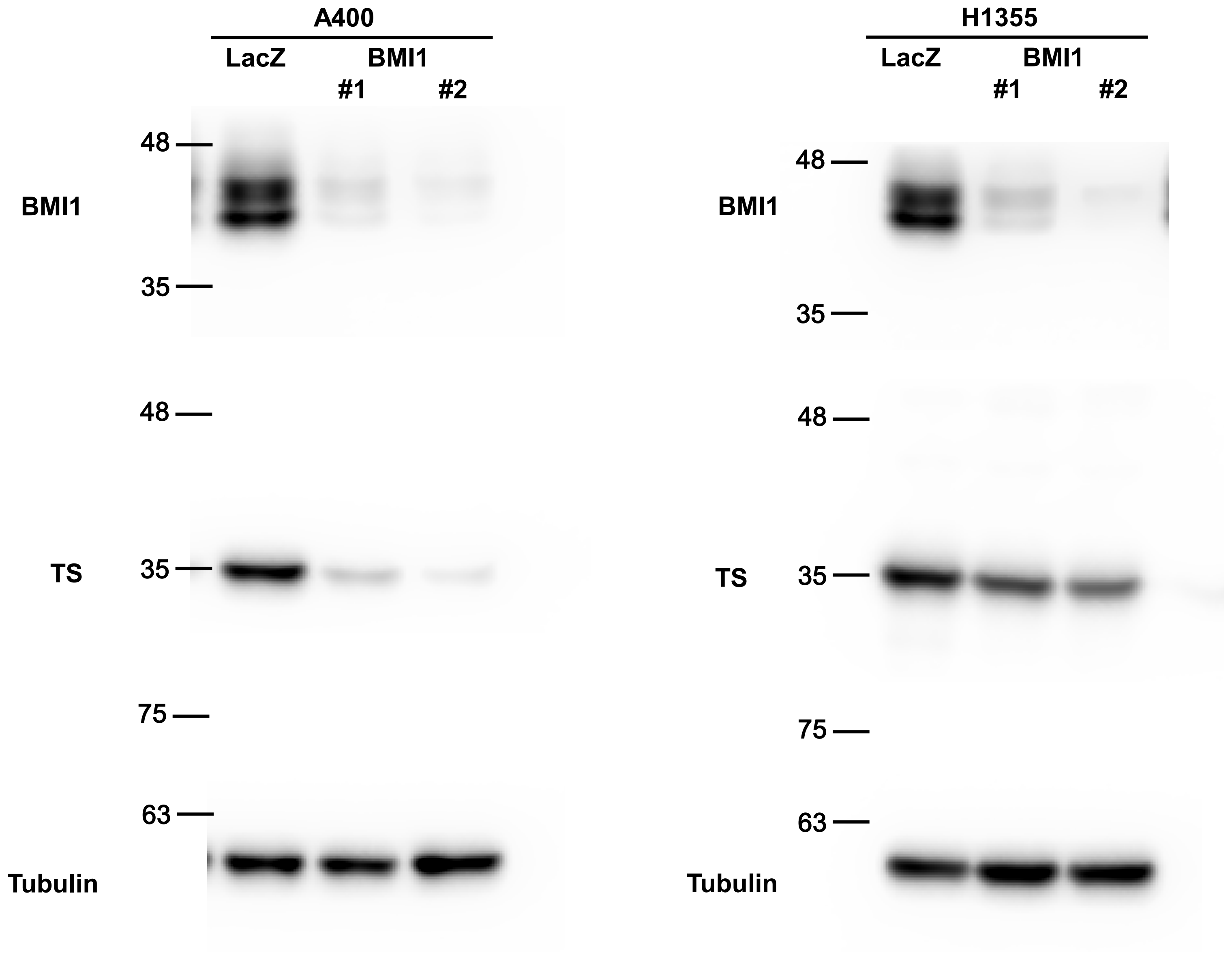
A400
LacZ
BMI1
#1
#2
48
BMI1
35
48
TS
35
75
63
Tubulin
H1355
LacZ
BMI1
#1
#2
48
BMI1
35
48
TS
35
75
63
Tubulin

Figure 5A.

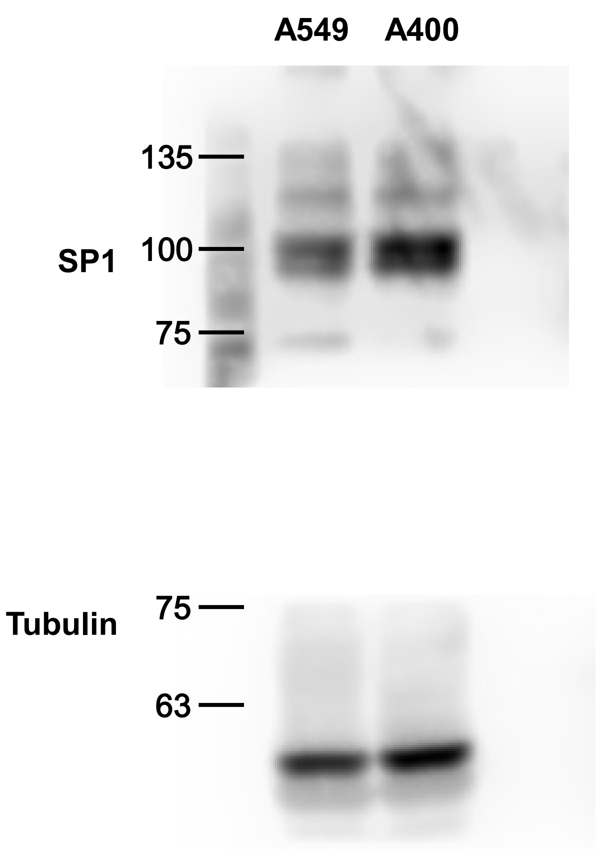

Figure 5B.

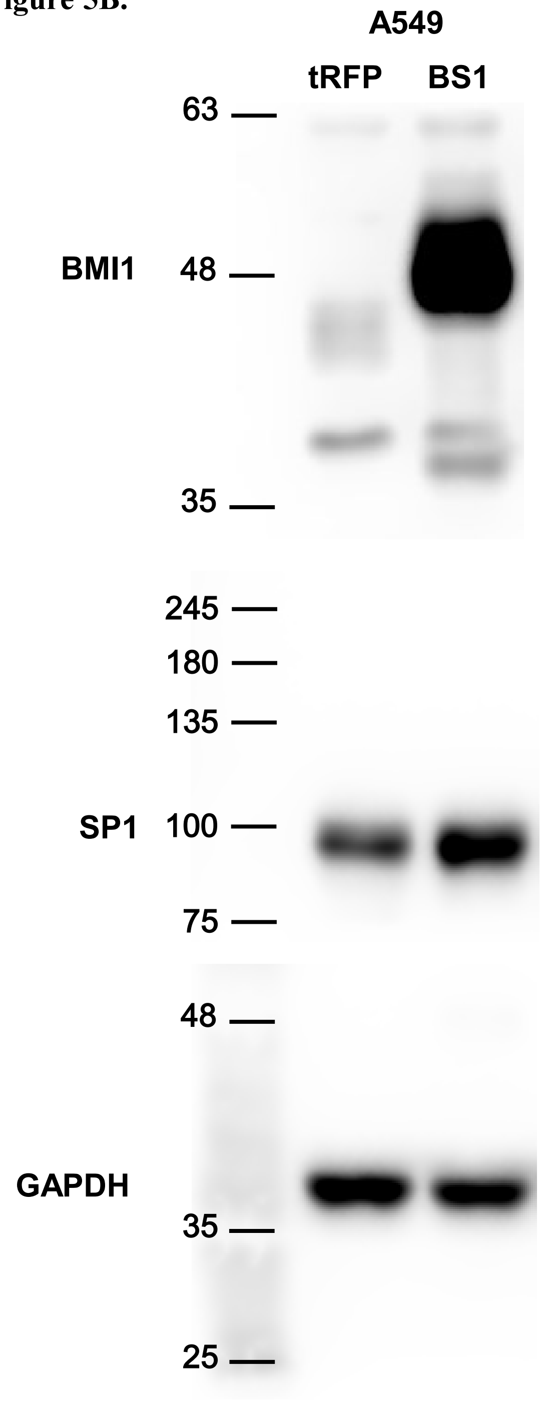

Figure 5D.

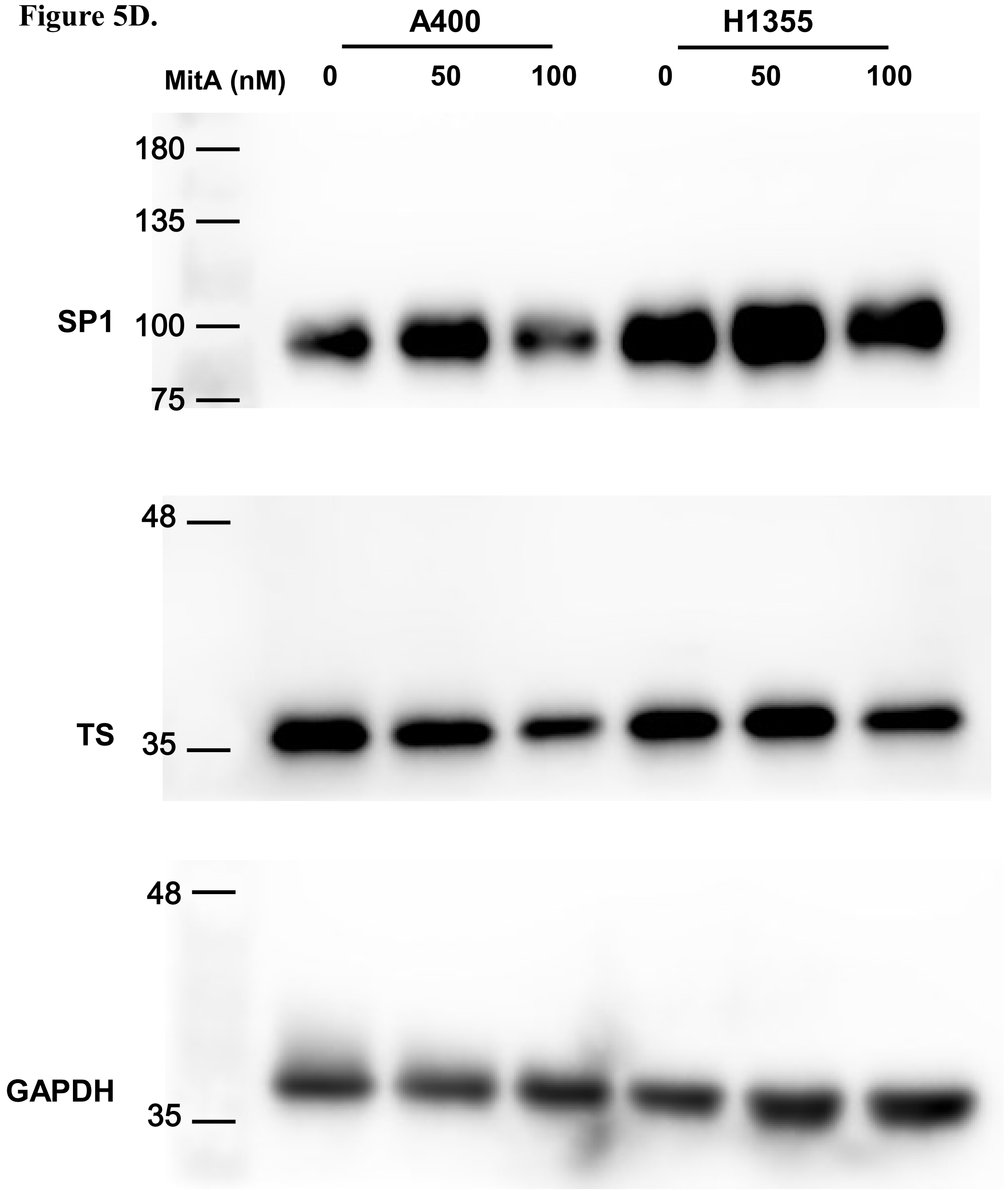

Figure 5E.

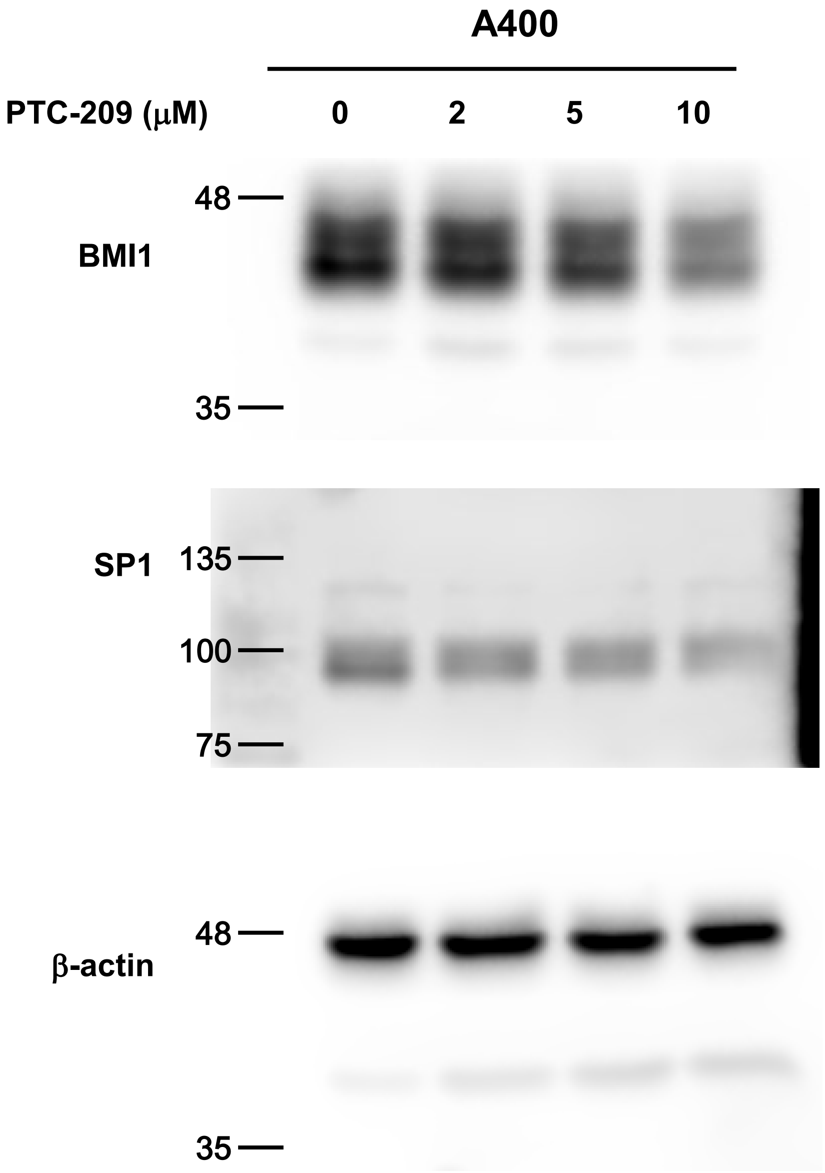

Figure 6A.

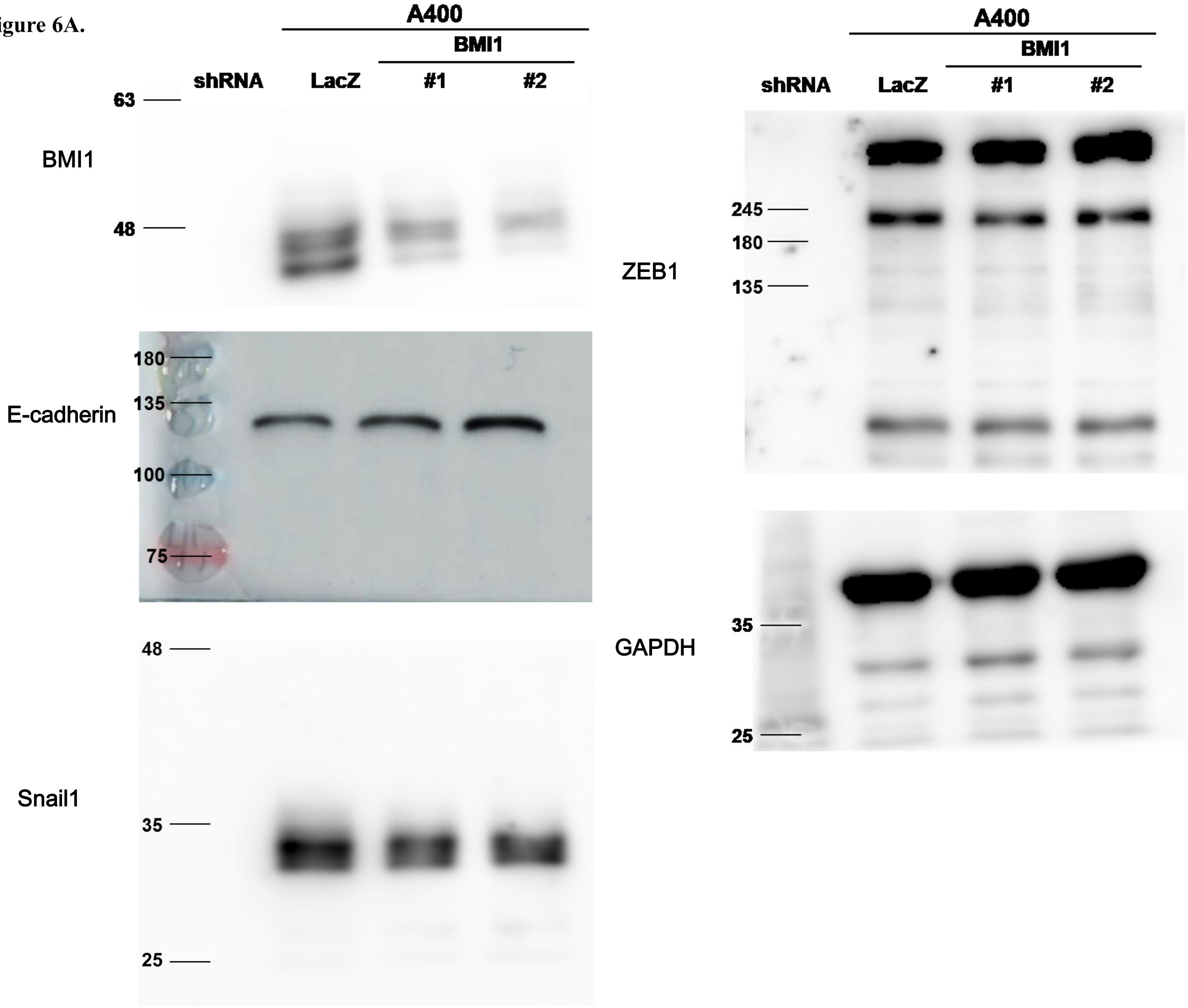
A400
BMI1
shRNA
LacZ
#1
#2
63
48
BMI1
180
135
100
75
E-cadherin
48
35
25
Snail1
A400
BMI1
shRNA
LacZ
#1
#2
245
180
135
ZEB1
35
25
GAPDH

Figure 6B.

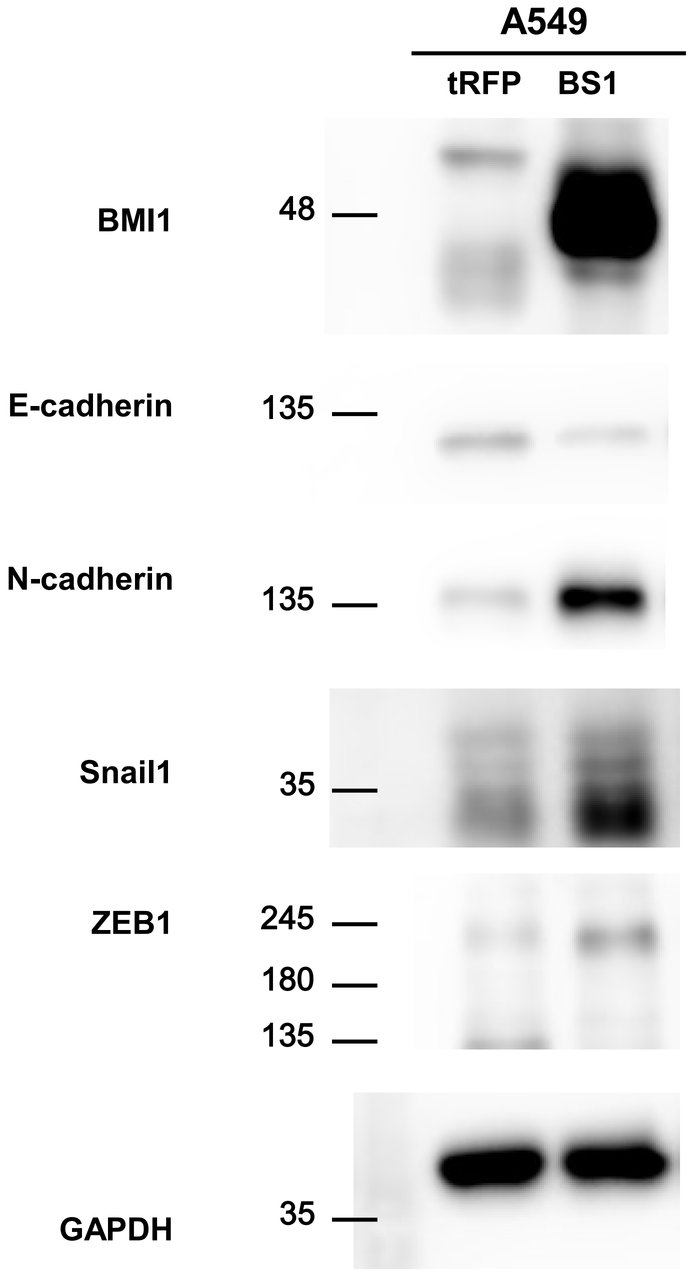
A549
tRFP
BS1
BMI1
48
E-cadherin
135
N-cadherin
135
Snail1
35
ZEB1
245
180
135
GAPDH
35

Figure S1.

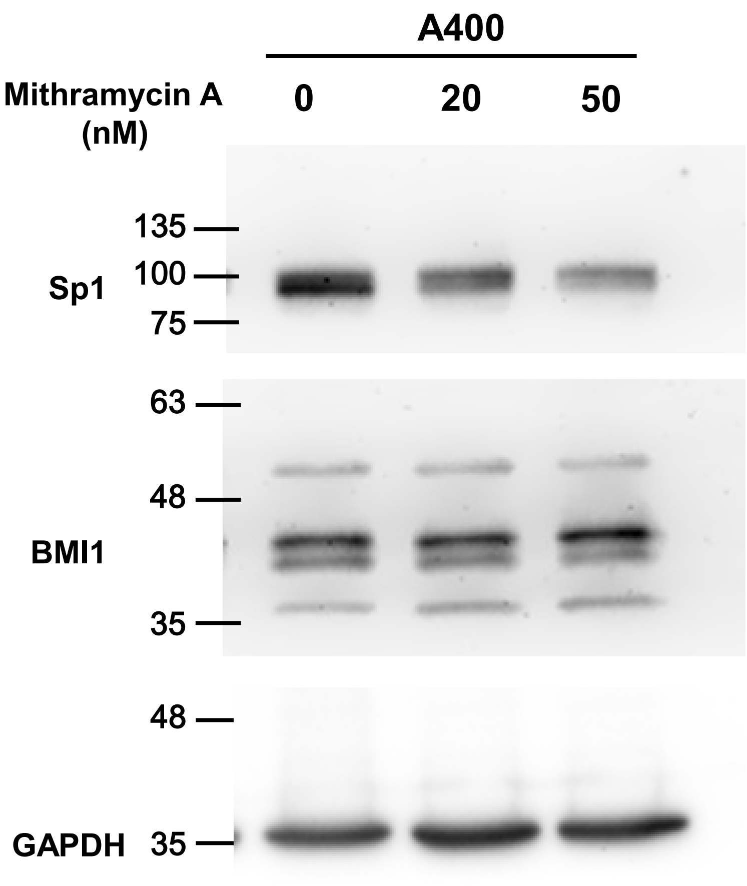
A400
Mithramycin A (nM)
0
20
50
135
100
75
Sp1
63
48
BMI1
35
48
GAPDH
35
